# Displayed and encoded antigens on adenovirus vectors optimize humoral and cellular immune responses in rhesus macaques

**DOI:** 10.1128/jvi.02152-25

**Published:** 2026-04-14

**Authors:** Zhenyu Li, Matthew D. J. Dicks, Louisa M. Rose, Rebecca A. Russell, Katherine McMahan, Ninaad Lasrado, Joseph Nkolola, Erica Borducchi, Jinyan Liu, Jayeshbhai Chaudhari, Sumi Biswas, Dan H. Barouch

**Affiliations:** 1 Center for Virology and Vaccine Research, Beth Israel Deaconess Medical Center 1859 , Boston, Massachusetts, USA; 2 SpyBiotech Limited 733966 , Oxford, United Kingdom; 3 The Jenner Institute, University of Oxford 6396, Oxford, United Kingdom; Dartmouth College Geisel School of Medicine, Hanover, New Hampshire, USA

## LETTER

Adenoviruses (Ads) have been widely studied for their potential to be used as vectors to express transgenes of interest for gene therapy and vaccines. During the COVID-19 pandemic, several Ad-based vaccines were utilized globally (1–5). Approximately 200 million people received the Johnson & Johnson Ad26.COV2.S vaccine globally, and 2 billion people received the AstraZeneca ChAdOx1 vaccine (4). Ad-based vaccines are typically made replication-defective by deleting E1 (5, 6). E3 can also be deleted to increase transgene capacity (7, 8), and a transgene of interest can be inserted into the viral genome.

Antigen display on nanoparticle scaffolds using the SpyTag/SpyCatcher system (9) has been demonstrated with various vaccine platforms, such as hepatitis B virus and mi3 nanoparticles (8, 10, 11). Recent work has shown that a related system, Dogtag/Dogcatcher (12), can be used to display immunogens on the capsid surface of adenovirus serotype 5 (Ad5) vectors, effectively utilizing the Ad capsid as the scaffold for a nanoparticle vaccines (13). DogTag (23 amino acids) was genetically inserted into surface-exposed loops in the adenovirus hexon capsid protein to allow covalent attachment of antigens fused to DogCatcher (a 15 kDa protein domain) on virus particles. Attachment by isopeptide bond formation between DogTag and DogCatcher was achieved by co-incubation of antigen and adenovirus components. In mice, Ad5 decorated with the SARS-CoV-2 spike receptor binding domain (S^RBD^) protein induced over 10-fold higher SARS-CoV-2 neutralization titers compared to an undecorated Ad5 encoding a spike (S) transgene (12).

Ad-based vaccines expressing transgenes have been shown to elicit durable humoral and cellular immune responses in both preclinical and clinical studies (14). Compared with ferritin nanoparticle vaccines, Ad vectors elicited higher CD8^+^ T cell responses but lower antibody responses in nonhuman primates (15). Here, we report the immunogenicity in nonhuman primates of Ad5 vectors that both encode SARS-CoV-2 spike (S) as a transgene and display spike receptor binding domain (S^RBD^) proteins on the capsid surface. This combined vaccine induced more robust humoral and cellular immune responses than Ad5 vectors that only encoded S transgene or only displayed S^RBD^ proteins.

**Fig 1:**
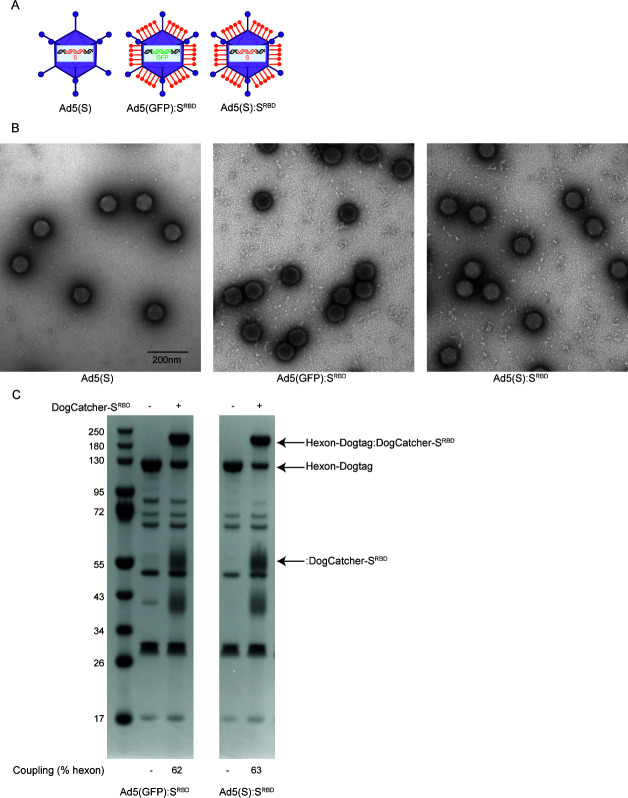
Ad5 vectors with or without S^RBD^. (**A**) Cartoons of vaccines. (**B**) Representative negative stain EM images of vaccines. Scale bar = 200 nm. (**C**) SDS-PAGE and Coomassie staining analysis of Ad5(GFP) or Ad5(S) virions, either undecorated or decorated with S^RBD^. Gel shift observed upon covalent coupling of DogCatcher-S^RBD^ to virion-associated hexon-DogTag. Coupling efficiencies based on quantitative densitometry are shown.

We conducted a head-to-head immunologic comparison of (i) Ad5 that encoded full-length WA1/2020 SARS-CoV-2 S as a transgene [Ad5(S)]; (ii) Ad5 that encoded green fluorescent protein (GFP) as a negative control transgene and that displayed S^RBD^ protein on the capsid surface [Ad5(GFP):S^RBD^]; and (iii) Ad5 that encoded S as a transgene and that displayed S^RBD^ protein on the capsid surface [Ad5(S):S^RBD^] (Fig. 1A). Negative stain electron microscopy showed no major differences in virus particle morphology between Ad5(S), Ad5(GFP):S^RBD^, and Ad5(S):S^RBD^ (Fig. 1B), and SDS-PAGE analysis with quantitative densitometry showed that the coupling efficiency of S^RBD^ to the hexon proteins was 62% and 63% for Ad5(GFP):S^RBD^ and Ad5(S):S^RBD^, respectively, similar to prior reports (Fig. 1C) (12).

**Fig 2:**
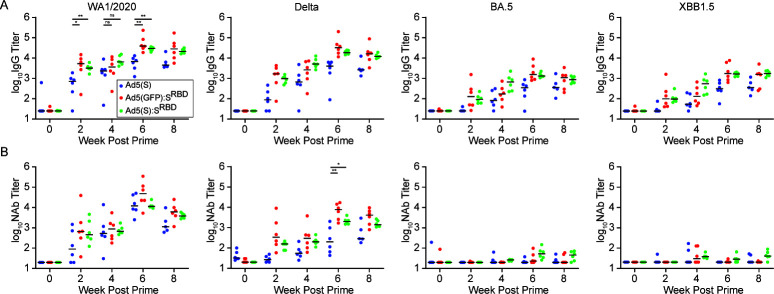
Displaying S^RBD^ on the Ad5 capsid results in higher binding and neutralizing antibody responses. (**A**) Binding antibody titers against SARS-CoV-2 WA1/2020, Delta, BA.5, and XBB1.5 at weeks 0, 2, 4, 6, 8 (*N* = 18, *n* = 6 per group). Animals were vaccinated at weeks 0 and 4. Median values are shown by the horizontal bars. (**B**) Neutralizing antibody titers against SARS-CoV-2 WA1/2020, Delta, BA.5, and XBB1.5 variants by luciferase-based pseudovirus neutralization assays. Median values are shown by the horizontal bars. Statistical analyses performed by Mann-Whitney U test, ∗*P* < 0.05; ∗∗*P* < 0.01; ∗∗∗*P* < 0.001; ns, not significant.

We immunized 18 rhesus macaques (*N* = 6/group) with 5 × 10^10^ viral particles (vp) of each of these vaccines by the intramuscular route at week 0 and week 4, and we measured humoral and cellular immune responses every 2 weeks. All animals developed SARS-CoV-2 WA1/2020, Delta, BA.5, and XBB.1.5 spike-specific binding antibodies by enzyme-linked immunosorbent assay (ELISA) by week 2 after the first immunization (Fig. 2A). By week 6, we observed a >10-fold increase in binding antibody titers in all groups. Median binding antibody titers against WA1/2020 spike at week 6 were 6,789, 39,261, and 29,873 for Ad5(S), Ad5(GFP):S^RBD^, and Ad5(S):S^RBD^, respectively. Consistent with a previous mouse study (12), Ad5(S):S^RBD^ and Ad5(GFP):S^RBD^ elicited 4.4-fold or 5.8-fold higher binding antibody titers than did Ad5(S), respectively, demonstrating the potency of the nanoparticle displayed S^RBD^ antigen for induction of humoral immunity (*P* = 0.0044, *P* = 0.0044 comparing binding antibody titers elicited by Ad5(S):S^RBD^ and Ad5(GFP):S^RBD^ to Ad5(S), respectively, at week 6 for WA1/2020).

Neutralizing antibody (NAb) titers at week 6 against SARS-CoV-2 Delta were 202, 7,717, and 1,985 for Ad5(S), Ad5(GFP):S^RBD^, and Ad5(S):S^RBD^, respectively (Fig. 2B). These data show that Ad5(S):S^RBD^ and Ad5(GFP):S^RBD^ elicited 10-fold or 38-fold higher NAb titers than Ad5(S), respectively (*P* = 0.0152 and *P* = 0.0086, respectively) (Fig. 2B). As expected, only low NAb responses were observed against BA.5 and XBB.1.5 with two immunizations of vaccines encoding WA1/2020 spike, as previously reported (16–18). Additionally, Ad5(GFP):S^RBD^ and Ad5(S):S^RBD^ elicited faster binding antibody kinetics than Ad5(S) from week 2 to week 4 (*P* = 0.0411, *P* = 0.0086, *P* = 0.5887, and *P* = 0.0513 at week 2 and 4, respectively). These results demonstrate that displaying S^RBD^ on the capsid surface results in both higher and faster antibody responses than transgene-encoded S only.

**Fig 3:**
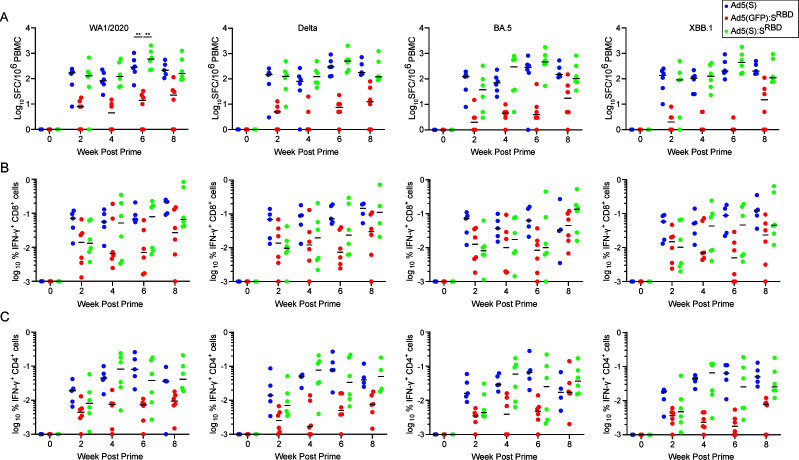
Encoding S as a transgene results in higher T cell response. (**A**) Spike-specific pooled peptide IFN-γ ELISpot responses to SARS-CoV-2 WA1/2020, Delta, BA.5, and XBB1.5 at weeks 0, 2, 4, 6, 8 (*N* = 18, *n* = 6 per group). Animals were vaccinated at weeks 0 and 4. Median values are shown by the horizontal bars. Spike-specific pooled peptide IFN-γ CD8^+^ T cells (**B**) and CD4^+^ T cells (**C**) were assessed by intracellular cytokine staining. Statistical analyses performed by Mann-Whitney U test, ∗*P* < 0.05; ∗∗*P* < 0.01; ∗∗∗*P* < 0.001; ns, not significant.

We next assessed spike-specific interferon-gamma (IFN-γ) cellular immune responses by pooled peptide enzyme-linked immunospot (ELISpot) and intracellular cytokine staining (ICS) assays. Ad5(S):S^RBD^ and Ad5(S) elicited higher ELISpot responses than did Ad5(GFP):S^RBD^. Median WA1/2020 spike-specific ELISpot responses were 283, 14, and 589 spot forming cells per million peripheral blood mononuclear cells (PBMC) for Ad5(S), Ad5(GFP):S^RBD^, and Ad5(S):S^RBD^ at week 6, respectively, demonstrating the potency of the encoded S transgene for induction of cellular immunity [*P* = 0.0044 and *P* = 0.0044 comparing WA1/2020 spike-specific ELISpot responses elicited by Ad5(S):S^RBD^ and Ad5(S) to Ad5(GFP):S^RBD^, respectively] (Fig. 3A) (12). Similar results were observed for both CD8^+^ and CD4^+^ T cell responses by ICS assays (Fig. 3B and C). Moreover, T cell responses were highly cross-reactive against multiple SARS-CoV-2 variants, consistent with previous reports (17, 19). These data show that the S transgene resulted in higher cellular immune responses than capsid displayed S^RBD^.

Our data show that display of S^RBD^ on an adenovirus capsid induced higher antibody responses, and that transgene-encoded S induced higher T cell responses in nonhuman primates. Moreover, the combined vaccine Ad5(S):S^RBD^ that both displays S^RBD^ on the capsid surface and encodes S as a transgene elicited both robust humoral and robust cellular immune responses. These data demonstrate that Ad5 can function simultaneously as a nanoparticle scaffold for a protein immunogen and as a classical viral vector for an encoded transgene. Future studies could evaluate the durability of these responses (20) and the immunogenicity of these vaccines in animals with baseline SARS-CoV-2 immunity. Future studies could also evaluate the potential of displaying B-cell immunogens (e.g., influenza virus hemagglutinin) on the capsid surface and encoding T cell immunogens (e.g., influenza virus matrix or nucleoprotein) as a transgene. These data suggest that adenovirus capsid decoration and transgene delivery have the potential to be a highly versatile and customizable platform, with applications in seasonal vaccination, pandemic preparedness, and personalized medicine.

## MATERIALS AND METHODS

### Animals

Eighteen outbred male and female rhesus macaques were housed at Alphagenesis, Yemassee, SC, USA. Animals were randomly allocated to experimental groups. Each animal was immunized with 5 × 10^10^ vp of each vaccine at week 0 and week 4. Blood samples were collected every 2 weeks for the first 2 months of the study and every 4 weeks for the remainder of the study.

### Vaccines

Construction of vaccines has been described previously (12). Briefly, WA1/2020 spike or GFP was inserted into E1/E3-deleted Ad5 after a cytomegalovirus immediate-early promoter containing a tetracycline operator sequence as previously described. DogTag (DIPATYEFTDGKHYITNEPIPPK) flanked by GSGGSG linkers was inserted into the hexon hypervariable region 5 (HVR5) loop through bacterial artificial chromosome galactokinase recombineering (12). Viruses were rescued and amplified in E1-complementing human embryonic kidney (HEK) 293 cell lines, either HEK293A (GFP-expressing Ad) or HEK293TREX (Invitrogen) (S-expressing Ad). Viruses were purified via CsCl gradient ultracentrifugation. Purified virus was stored in sucrose storage buffer (10 mM Tris-HCl, 7.5% wt/vol sucrose, pH 7.8) at −80°C. SARS-CoV-2 spike receptor binding domain (RBD) fused to dogcatcher (Dogcatcher-S^RBD^) (WA1/2020, residues 319–532) was cloned into a mammalian protein expression plasmid, produced in ExpiCHO (Chinese hamster ovary)-S cells, and purified via C-tag affinity chromatography (Thermo Fisher) as previously described (12). Purified proteins were dialyzed into tris-buffered saline. DogTag/DogCatcher conjugation to attach DogCatcher-S^RBD^ to adenovirus particles was performed as previously described (12). Excess ligand was removed by SpectraPor dialysis cassettes (300-kDa MWCO), and decorated particles were stored at −80°C (12). All vaccines were filter sterilized (0.22 µm, Millex-GP) prior to storage.

### Assessing coupling efficiency

The S^RBD^ coupling efficiency was assessed by SDS-PAGE and quantitative densitometry as previously described (12). Briefly, coupled and uncoupled samples were boiled at 95°C for 5 min before loading onto SDS-PAGE (NuPAGE 4%–12% Bis-Tris, Invitrogen). Proteins were resolved at 220 V for 55 min. The gel was stained by Coomassie staining for 16 h and destained with water. Coupling efficiency was assessed by comparing band intensity using densitometry with ImageJ.

### Negative-stain electron microscopy

Vaccines were analyzed by negative stain electron microscopy (EM). Five microliters of sample (20 µg/mL) was applied to a carbon-coated copper grid (EMS; CF400-CU) that had been glow discharged for 20 s at 25 mA to render the surface hydrophilic. After 1 min of adsorption, excess liquid was removed with filter paper (Whatman No. 1). The grid was briefly floated on a drop of distilled water to remove residual phosphate or salt, blotted, and stained with 0.75% (weight/volume) uranyl formate (EMS; catalog no. 22451) for 20 s. Excess stain was removed with filter paper, and the grids were examined using a JEOL JEM120i transmission electron microscope at 40,000× magnification, and images were recorded with an Advanced Microscopy Techniques NanoSprint 15-MKII camera.

### Enzyme-linked immunosorbent assay

SARS-CoV-2 spike-specific binding antibodies in serum were assessed by ELISA. 96-well plates were coated with 1 μg/mL of similarly produced SARS-CoV-2 WA1/2020, Delta, BA.5, or XBB1.5 spike protein receptor binding domain in 1× Dulbecco phosphate-buffered saline (DPBS) and incubated at 4°C overnight. After incubation, plates were washed once with wash buffer (0.05% Tween 20 in 1× DPBS) and blocked with 350 μL of casein block solution per well for 2 to 3 h at room temperature. Following incubation, the block solution was removed, and the plates were blotted dry. Heat-inactivated serum was serially diluted in casein block buffer containing wells. Plates were incubated at room temperature for 1 h, prior to three more washes and a 1-h incubation with a 1:1,000 dilution of anti-macaque IgG horseradish peroxidase (Nonhuman Primate Reagent Resource) at room temperature in the dark. Plates were washed three more times, and 100 μL of SeraCare KPL TMB SureBlue Start solution was added to each well; plate development was halted by adding 100 μL of SeraCare KPL 3,3′,5,5′-tetramethylbenzidine (TMB) Stop solution per well. The absorbance at 450 nm, with a reference at 650 nm, was recorded with a VersaMax microplate reader (Molecular Devices). For each sample, the ELISA endpoint titer was calculated using a 4-parameter logistic curve fit to calculate the reciprocal serum dilution that yields an absorbance value of 0.2. Interpolated endpoint titers were reported.

### Pseudovirus neutralization assay

Heat-inactivated sera were tested for neutralization against WA1/2020, Delta, BA.5, and XBB1.5 SARS-CoV-2 variants as previously described using lentivirus-based pseudotyped virus encoding a luciferase reporter gene. Briefly, human embryonic kidney HEK293T cells (ATCC CRL_3216) were co-transfected with a luciferase reporter plasmid (pLenti-CMV Puro-Luc, Addgene), packaging construct psPAX2 (AIDS Resource and Reagent Program), and spike protein expressing pcDNA3.1-SARS-CoV-2 SΔCT using Lipofectamine 2000 (Thermo Fisher). The pseudovirus-containing supernatants were collected 2 days after transfection and filtered through a 0.45-μm filter. To determine neutralization titers, threefold dilutions of the sera were pre-incubated with the same volume of pseudovirus for 1 h at 37°C before the sera/pseudovirus mix was transferred to HEK293T-Angiotensin-converting enzyme 2 (ACE2) cells seeded the day before (2 × 10^4^ cells/well). Approximately 48 h later, cells were lysed using Steady-Glo Luciferase System (Promega) according to the manufacturer’s instructions. Neutralization titer was determined as the sera dilution in which 50% of relative light units were observed, when 100% relative light units were set to be virus only, and 0% were set to be with neither virus nor sera.

### ELISpot assay

Peptide pools were 15-amino acid peptides overlapping by 11 amino acids spanning the SARS-CoV-2 WA1/2020, Delta, BA.5, or XBB1.5 spike proteins (21st Century Biochemicals). IFN-γ ELISpot was performed on PBMCs. ELISpot plates were coated with mouse anti-human IFN-γ monoclonal antibody from BD Pharmingen at 0.5 µg per well and incubated overnight at 4°C. Plates were washed with DPBS containing 0.25% Tween 20 and blocked with R10 media (500 mL Roswell Park Memorial Institute 1640 [RPMI 1640] with 55 mL fetal bovine serum [FBS] and 5.5 mL of 100× penicillin-streptomycin with a final concentration of 100 units/mL penicillin and 100 ug/mL streptomycin) for 1 to 4 h at 37°C. SARS-CoV-2 pooled S peptides (1 µg/mL) and cells (2 × 10^5^/well) were added to the plate and incubated for 18 to 24 h at 37°C. All steps following this incubation were performed at room temperature. The plates were washed with Coulter buffer and incubated for 2 h with biotinylated anti-human IFN-γ antibody from U-CyTech Biosciences (1 µg/mL in each well). The plates were washed a second time and incubated for 2 h with streptavidin-alkaline phosphate (AP) antibody from Southern Biotechnology (2 µg/mL in each well). The final wash was followed by the addition of warmed and filtered nitro blue tetrazolium chloride/5-bromo-4-chloro-3-indolyl phosphate p-toluidine salt (BCIP/NBT chromogen) substrate solution from Pierce. The chromogen was discarded, and the plates were washed with water and dried out of direct light for 24 h. Plates were scanned and counted on a KS ELISpot Reader. Results are expressed with background subtraction.

### Intracellular cytokine staining assay

CD4+ and CD8+ T cell responses were quantitated by pooled peptide-stimulated ICS assays. Peptide pools were the same as mentioned above in the ELISpot assay. A total of 10^6^ peripheral blood mononuclear cells wells were re-suspended in 100 μL of R10 media supplemented with CD49d monoclonal antibody (1 μg/mL) and CD28 monoclonal antibody (1 μg/mL). Each sample was assessed with mock (100 μL of R10 plus 0.5% dimethyl sulfoxide; background control), peptides (2 μg/mL), and/or 10 pg/mL phorbol myristate acetate and 1 μg/mL ionomycin (Sigma-Aldrich) (100 μL; positive control) and incubated at 37°C for 1 h. After incubation, 0.25 μL of GolgiStop and 0.25 μL of GolgiPlug in 50 μL of R10 were added to each well and incubated at 37°C for 8 h and then held at 4°C overnight. The next day, the cells were washed twice with DPBS, stained with aqua live/dead dye for 10 min, and then stained with predetermined titers of monoclonal antibodies against CD279 (clone EH12.1, BB700), CD38 (clone OKT10, PE), CD28 (clone 28.2, PE CY5), CD4 (clone L200, BV510), CD95 (clone DX2, BUV737), CD8 (clone SK1, BUV805) for 30 min. Cells were then washed twice with 2% FBS/DPBS buffer and incubated for 15 min with 200 μL of BD CytoFix/CytoPerm Fixation/Permeabilization solution. Cells were washed twice with 1× Perm Wash buffer (BD Perm/WashTM Buffer 10× in the CytoFix/CytoPerm Fixation/Permeabilization kit diluted with MilliQ water and passed through 0.22 μm filter) and stained intracellularly with monoclonal antibodies against Ki67 (clone B56, FITC), CD69 (clone TP1.55.3, ECD), IL10 (clone JES3-9D7, PE CY7), IL13 (clone JES10-5A2, BV421), TNF-α (clone Mab11, BV650), IL4 (clone MP4-25D2, BV711), IFN-γ (clone B27; BUV395), CD45 (clone D058-1283, BUV615), IL2 (clone MQ1-17H12, APC), CD3 (clone SP34.2, Alexa 700) for 30 min. Cells were washed twice with 1× Perm Wash buffer and fixed with 250 μL of freshly prepared 1.5% formaldehyde. Fixed cells were transferred to 96-well round-bottom plates and analyzed by BD FACSymphony system. Data were analyzed using FlowJo v9.9.

### Statistical analysis

Data were plotted and analyzed by GraphPad Prism v10.6.1. All statistical analyses were compared using two-sided Mann-Whitney U-tests with Holm-Bonferroni correction. *P* < 0.05 was considered to be significant.

## Data Availability

All data are available in the paper and from D.H.B. (dbarouch@bidmc.harvard.edu).
